# Can We Convert Genotype Sequences Into Images for Cases/Controls Classification?

**DOI:** 10.3389/fbinf.2022.914435

**Published:** 2022-06-28

**Authors:** Muhammad Muneeb, Samuel F. Feng, Andreas Henschel

**Affiliations:** ^1^ Department of Mathematics, Khalifa University of Science and Technology, Abu Dhabi, United Arab Emirates; ^2^ Department of Electrical Engineering and Computer Science, Khalifa University of Science and Technology, Abu Dhabi, United Arab Emirates; ^3^ Research and Data Intelligence Support Center R-DISC, Khalifa University of Science and Technology, Abu Dhabi, United Arab Emirates

**Keywords:** genotype-phenotype prediction, genetics, bioinformatics, applied machine learning, image classification

## Abstract

Converting genotype sequences into images offers advantages, such as genotype data visualization, classification, and comparison of genotype sequences. This study converted genotype sequences into images, applied two-dimensional convolutional neural networks for case/control classification, and compared the results with the one-dimensional convolutional neural network. Surprisingly, the average accuracy of multiple runs of 2DCNN was 0.86, and that of 1DCNN was 0.89, yielding a difference of 0.03, which suggests that even the 2DCNN algorithm works on genotype sequences. Moreover, the results generated by the 2DCNN exhibited less variation than those generated by the 1DCNN, thereby offering greater stability. The purpose of this study is to draw the research community’s attention to explore encoding schemes for genotype data and machine learning algorithms that can be used on genotype data by changing the representation of the genotype data for case/control classification.

## 1 Introduction

The genotype sequence Nielsen et al. (2011) is a linear sequence of bases (A, C, G, and T) in a DNA molecule, interpreted as homozygous or heterozygous and represented as 0,1,2 (encoding) for numerical analysis. These sequences can also be expressed in other forms depending on the problem statement, such as genotype-phenotype classification, genotype sequence similarity, and dimensionality reduction. Similarly, there are various algorithms for each problem, like PRS (polygenic risk scores) for disease prediction, BLAST Madden et al. (2018) for sequence similarity, and principal component analysis (PCA) Salem and Hussein (2019) for dimensionality reduction. We can also use algorithms developed for similar problem statements, such as genotype sequences can be treated as documents, and document classification can be used for genotype-phenotype classification. In this study, we investigated the question, ‘Can we convert genotype sequences in the form of images for cases/control classification?’

Following is the list of acronyms used in the remaining sections of this study.

• CNN: Convolutional Neural Network
• ANN: Artificial Neural Network (fully connected)
• 1DCNN: One-dimensional Convolutional Neural Network
• 2DCNN: Two-dimensional Convolutional Neural Network
• CEU: Utah Residents (CEPH) with Northern and Western European ancestry (European population)

There are several reasons for doing this, which we will explore in this section. First, for image classification, a vast amount of work has been done by researchers (image classification, image segmentation, and finding similar images Appalaraju and Chaoji (2017)), and several image classification algorithms (CNN) can outperform ANN for a few tasks such as image recognition. We investigated, ‘Can we use CNN for genotype sequences?’ Well, yes, and researchers have used 1DCNN for genotype sequence classification Muneeb and Henschel (2021), but what about the 2DCNN algorithm, which requires genotype data in the form of images. Suppose the 2DCNN algorithm is successful in case/control classification. In this case, we can use all (visualize filters and feature maps) other image analysis algorithms for genotype sequences. For instance, we can identify the regions in images that make a particular person’s case and control. The above statements may not be biologically plausible because when we use the 2DCNN algorithm, SNPs that are not near can be part of one CNN kernel.

Second, genotype sequences are usually very long and require a dimensionality reduction algorithm (PCA) or statistical method (p-value thresholding) to make it feasible to train the machine learning model. When genotype sequences are converted to images, various encoding schemes can reduce the size of genotype data, as shown in this Section 3.5. Consider an image with 1024 by 1024. The number of pixels in the image is 1024 * 1024 = 1,048,576, which means 1,048,576 SNPs can be included in one image (image-size reduction algorithms can also be used to reduce the size). In the literature, many algorithms have been used to systematically reduce data sizes, such as data compression algorithms or encryption algorithms; however, researchers have not applied these algorithms to genotype sequences, which can generate exciting results.

We can also visualize how the homozygous and heterozygous rates change across the DNA sequences. The most crucial factor is that information from all SNPs is incorporated to classify cases/controls rather than using a single SNP at a time. There are other functions like sequence comparison, which can be performed on images to determine the similarity between two images Hoang et al. (2016).

The following text explains the rationale for using specific tools in the methodology section (see Section 3). We used Hapgen2 to generate genotype data because it allows the use of 1000 Genome + plus Hapmap3 CEU data to generate data (see 7, Dataset 1). Although we considered chromosome 21, any chromosome could be used for analysis. We used PhenotypeSimulator to generate phenotypes for each person, a rich tool that provides many options to produce variations in the phenotype, such as the number of risk SNPs and genetic variations.

## 2 Related Work

Researchers have already used algorithms like ANN, 1DCNN, and LSTM for genotype-phenotype prediction, but we did not find anyone using 2DCNN for genotype data after converting it into images.

Researchers employed variations of 1DCNN (one dimensional convolutional neural networks) for genotype-phenotype prediction Ma et al. (2018), Washburn et al. (2021), Jeong et al. (2020), Jubair and Domaratzki (2019), Onimaru et al. (2020), and Abdollahi-Arpanahi et al. (2020). This article Ma et al. (2018) used 1DCNN to reduce the complexity of genotype data and for phenotype prediction. We used 2DCNN for phenotype prediction and p-values threshold to reduce the number of SNPs passed to 2DCNN. The article claims that 1DCNN can be combined with the existing approaches for the prediction. Similarity genotype data can be converted to images of various dimensions to train an ensemble model for the prediction.

In this article Washburn et al. (2021), researchers used genetic, environmental, management, and historical information for phenotype prediction. In contrast, we just used the genetic information because we worked on the simulated data, and there is no environmental (covariates: sex and gender) information available. In this article Jeong et al. (2020), researchers used the GWAS-based SNPs selection method and used 1DCNN for optimal prediction. The report focused on improved marker selection, and our work is related to marker visualization by visualizing the convolutional layers of the trained 2DCNN model.

In this article Pérez-Enciso and Zingaretti (2019), researchers performed 1DCNN hyperparameter optimization and a survey of papers that compared machine learning with statistical tools. In Waldmann et al. (2020), researchers used 1DCNN combined with l1-norm regularization, Bayesian optimization, and ensemble prediction. In Chen and Shi (2019), researchers used 1DCNN and sparse autoencoder for genotype-phenotype prediction. This study Liu et al. (2019), uses the dual 1DCNN stack for classification. This study Yin et al. (2018), is analogous to ours and employs Hilbert curves to ensure that the pixels representing two sequence components that are near within the sequence are likewise close within the image. In Poplin et al. (2018), researchers used CNN to call genetic variations in aligned next-generation sequencing read data.

## 3 Methodology

**FIGURE 1:**
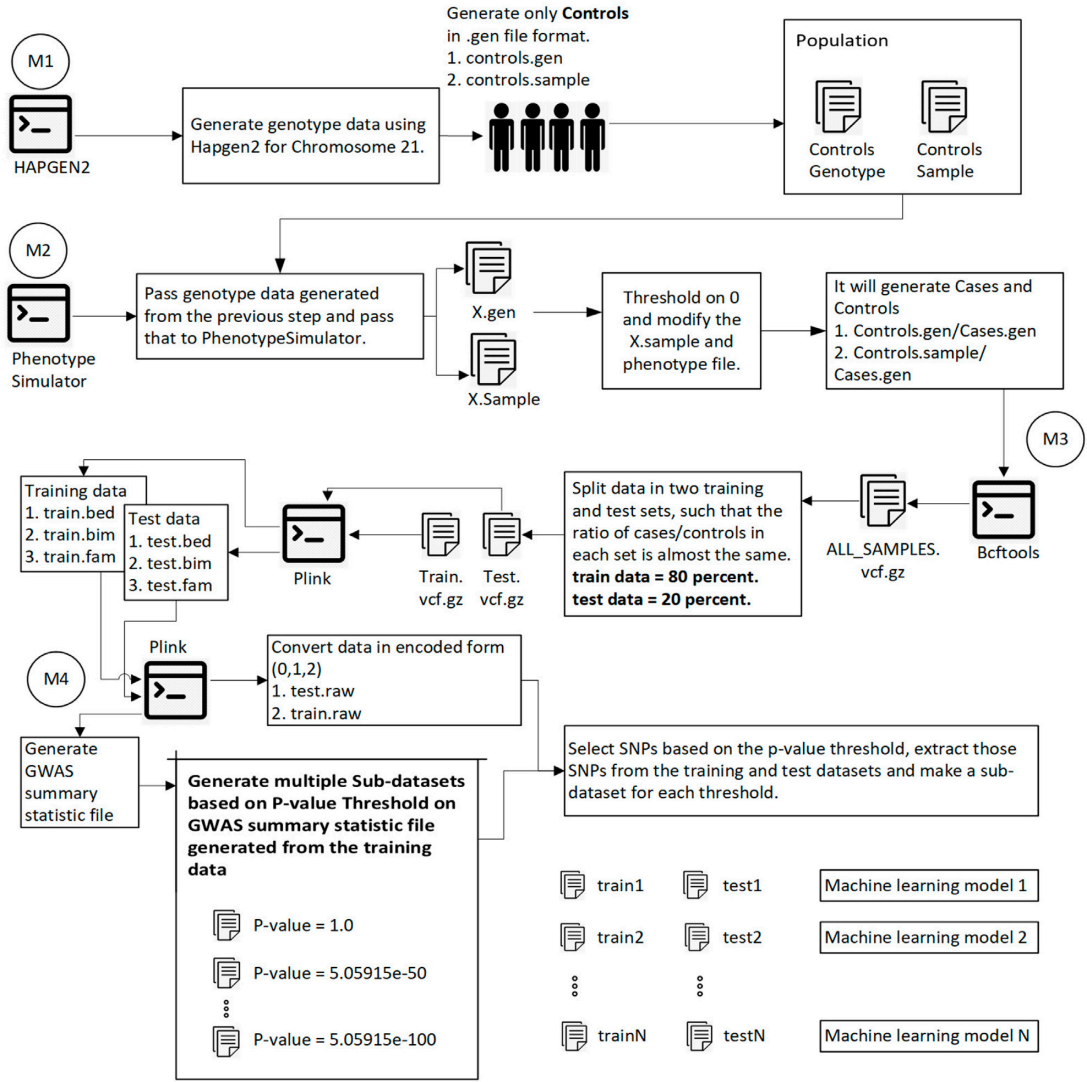
This diagram shows the overall process of generating data, splitting data into training and test sets, calculating the p-value for each SNP, and generating sub-datasets based on p-value thresholding. Module 1: Pass 1000 Genome and hapmap3 datasets (Chromosome 21) to hapgen2 and generate 10,000 controls (gen and controls.sample files). Module 2: Pass previously generated data to PhenotypeSimulator, which produces each person’s phenotype. Convert continuous phenotype to binary phenotype (cases/controls) by thresholding at 0. Module 3: Merge all cases/controls and convert the data in. vcf.gz file format. Split the data into training (80%) and test data (20%) such that the ratio of cases/controls in each set is the same. Using plink convert train.vcf.gz and test. vcf.gz to plink file format (.bed,.bim,.fam). Module 4: Using plink, generate a GWAS summary statistic file that contains the p-value for each SNP. Extract SNPs based on p-value threshold from training and test set and recode the genetic information (aa = 0, aA//Aa = 1, AA = 2). We have the training and test data ready to be converted into images at this step.

This section explores the dataset, methodology, intermediate steps, and machine learning algorithm used for the analysis. Figure 1 shows the overall methodology.

### 3.1 Dataset

Using hapgen, which takes 1000 Genome and HapMap3 data (Resource O, 2021) as input resource, we generated 10,000 (D1) controls for chromosome 21 and passed that to phenotypesimulator (PS) to generate cases/controls. The phenotype generated by PS for each person is continuous, which we converted to binary phenotype by thresholding on 0.

The sequence reads and library size does not impact the genotype data generation process. We used the 1000 Genome + HapMap3 dataset for the sequence generation. After that, we used Hapgen2 to generate the genotype data. This generates the data for all the SNPs in the original data. Hapgen2 inherits all the properties of the original data. Where the genotype data is missing, it imputes the missing information for all the people such that the linkage disequilibrium pattern is intact. PhenotypeSimulator does not modify the genotype data but only generates the phenotype for a particular person.

### 3.2 Dataset Split

**FIGURE 2:**
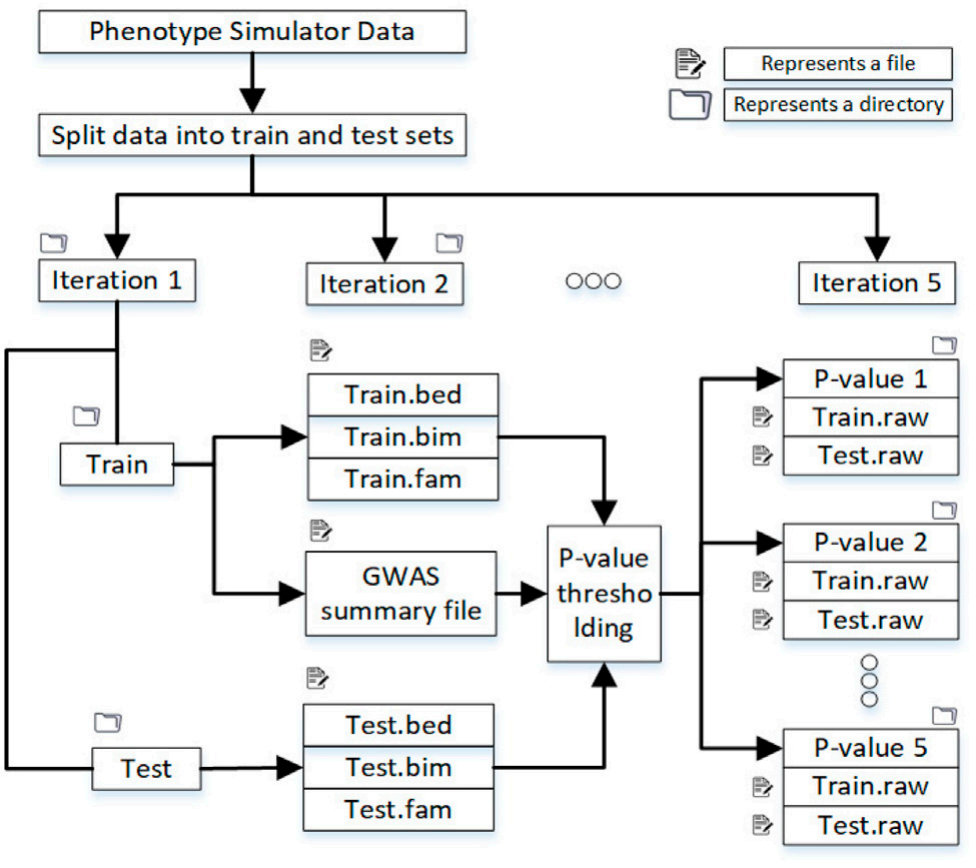
Split the dataset into training and test sets, and in each iteration, samples are randomly selected. After the p-value threshold, selected SNPs are extracted from the training and test data.

The training data was 80 percent, and the test data was 20 percent of the original datasets containing almost the same number of cases and controls. Figure 2 shows the directory structure in which files to train the machine learning model are produced.

### 3.3 Generate GWAS Summary Statistic File

We used training data to calculate the GWAS summary statistic file, which contains the p-value required for the SNPs preselection process. The association test can be performed using the following command, where file train contains the training genotype data.

./plink –bfile ./train –allow-no-sex –fisher –out GWAS

### 3.4 SNPS Preselection

To reduce the number of SNPs, we used a p-value threshold. We considered four p-value thresholds for D1. We repeated the classification process five times, so the actual number of SNPs after p-value thresholding varies in each run and is shown in the result section.

### 3.5 Model and Implementation

We used 2DCNN (Architecture shown in Table 1) for training, but before that, genotype data should be converted to images. Figures 5, 6 show the encoding process. The machine learning model has many parameters, so to find the best parameters, we considered parameters for the first iteration and the best parameters (see Table 2) were used for the remaining iterations.

**TABLE 1:** Model 2 architecture for dataset 2.

CNN architecture for all datasets	
Layers	Parameters
Layer 1−Con2D	32 Filters * (kernel size = (3,3))
Layer 2−MaxPool2D	(pool size = (2,2))
Layer 3−Con2D	64 Filters * (kernel size = (3,3))
Layer 4−MaxPool2D	(pool size = (2,2))
Reshape	—
Layer 5−FullyConnected	(10 Neurons)
Activation Layer	—
Layer 6−FullyConnected	(2 Neurons)
Softmax	—

**TABLE 2:** Hyper-parameters for all models for all datasets are the same.

Model’s Hyper-parameters	
Hyper-parameters	Value
Batch size	100
Epochs	50
Validation size	0.3%
Optimizer	Adam
Dropout	0.3
Activation	Relu
Loss	Binary_crossentropy
Metrics	Accuracy

#### 3.5.1 Deep Learning Model

**FIGURE 3:**
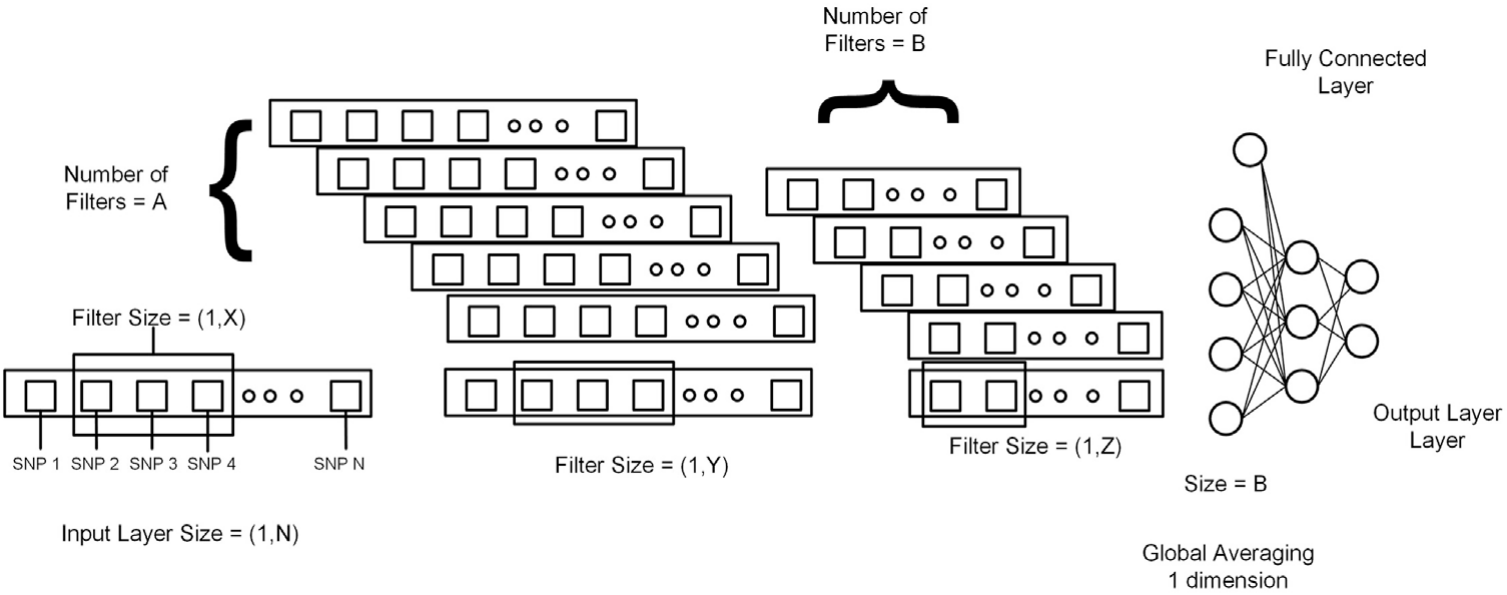
Genotype data is presented in the form of a sequence from 1 to N (N = Total number of SNPs). X, Y, and Z represent the filter size for the first layer, second layer, and third layer, respectively. A and B represent the number of filters in the first layer and second layer. At the end output of the last 1DCNN layer, after global averaging, is connected to the fully connected network.

**FIGURE 4:**
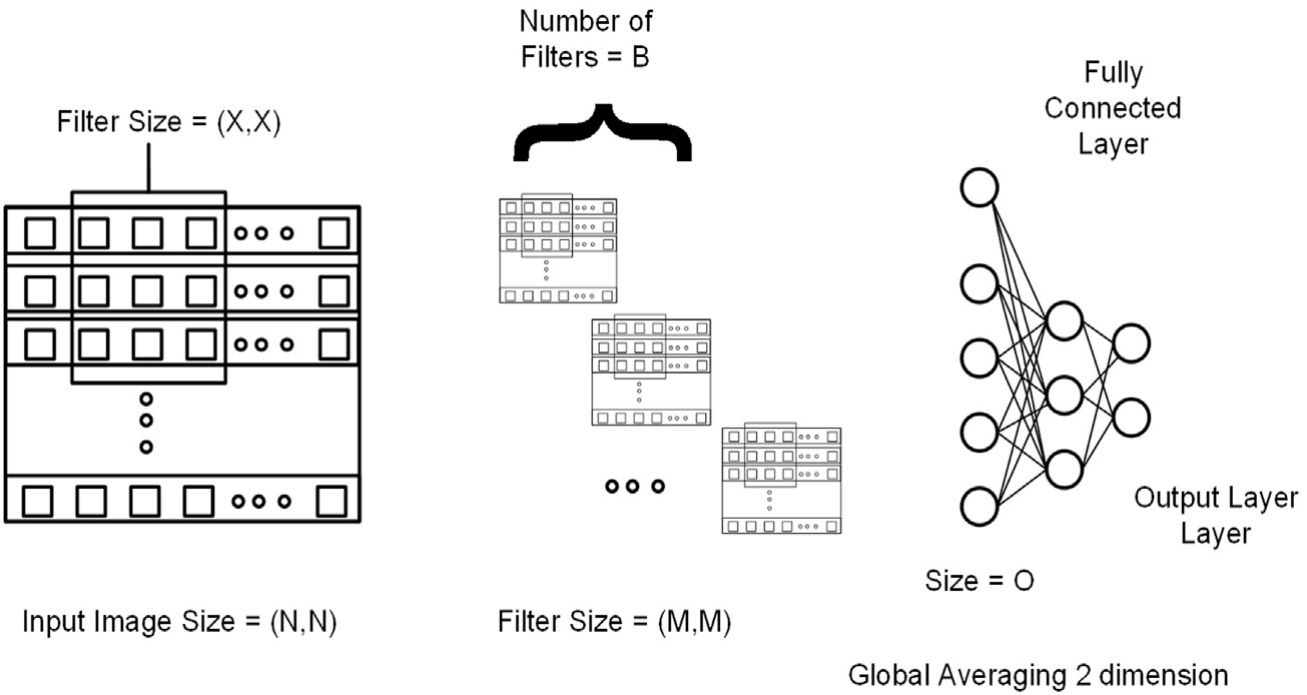
Genotype data is presented in the form of a sequence image having dimension N by N (N*N = Total number of SNPs). M and X represent the filter size for the first and second convolutional layers, respectively. B represents the number of filters in the second layer. At the end output of the last 2DCNN layer, after global averaging, is connected to the fully connected network.

This section explains 2DCNN and 1DCNN algorithms for this study. Figures 3, 4 show the architecture of the 1DCNN and 2DCNN models, respectively. The genotype data after p-value thresholding is converted to a linear sequence for the 1DCNN model. 1DCNN model uses a set of one-dimensional filters which extract useful information from the genotype data. 2DCNN model uses a set of two-dimensional filters to extract the information, so genotype data is converted into the form of images. 2DCNN algorithms can be trained on images of any dimension, and if the image dimension is X by Y where X = 1 and Y = N or X = N and Y = 1, the 2DCNN algorithm becomes the same as the 1DCNN algorithm. 2DCNN model can process images of any dimension, but we considered square images for simplicity, and the remaining SNPs were discarded.

#### 3.5.2 Image Encoding Schemes

This section explains the procedure to convert genotype data into images and possible encoding schemes before genotype data is passed to the 2DCNN algorithm.

In general, when we process DNA, there are four steps. 1. DNA sequencing (Read sequences) − > 2. Sequence alignment − > 3. DNA encoding for a particular application − > 4. Application.

In our case, this flow is transformed into the following sequence. 1. DNA sequencing (Read sequences) − > 2. Sequence alignment − > 3. AA = 0, AG/GA=1, GG =2 − > 4. Cases/controls classification.

A particular encoding scheme is beneficial for a particular application; for instance, phylogenetic trees are good for finding the evolutionary relationship among genes or species. The phylogenetic tree is the output of some analysis, and using it for the cases/controls classification is not possible. If we pass this tree as an input to 2DCNN, we have to construct it for each DNA sequence in the test set, which is impossible. Second, such trees can be used for classification (not cases/controls classification), but rather than using CNN; graph neural networks would work in such a situation because they are good at finding the relation between features.

**FIGURE 5:**
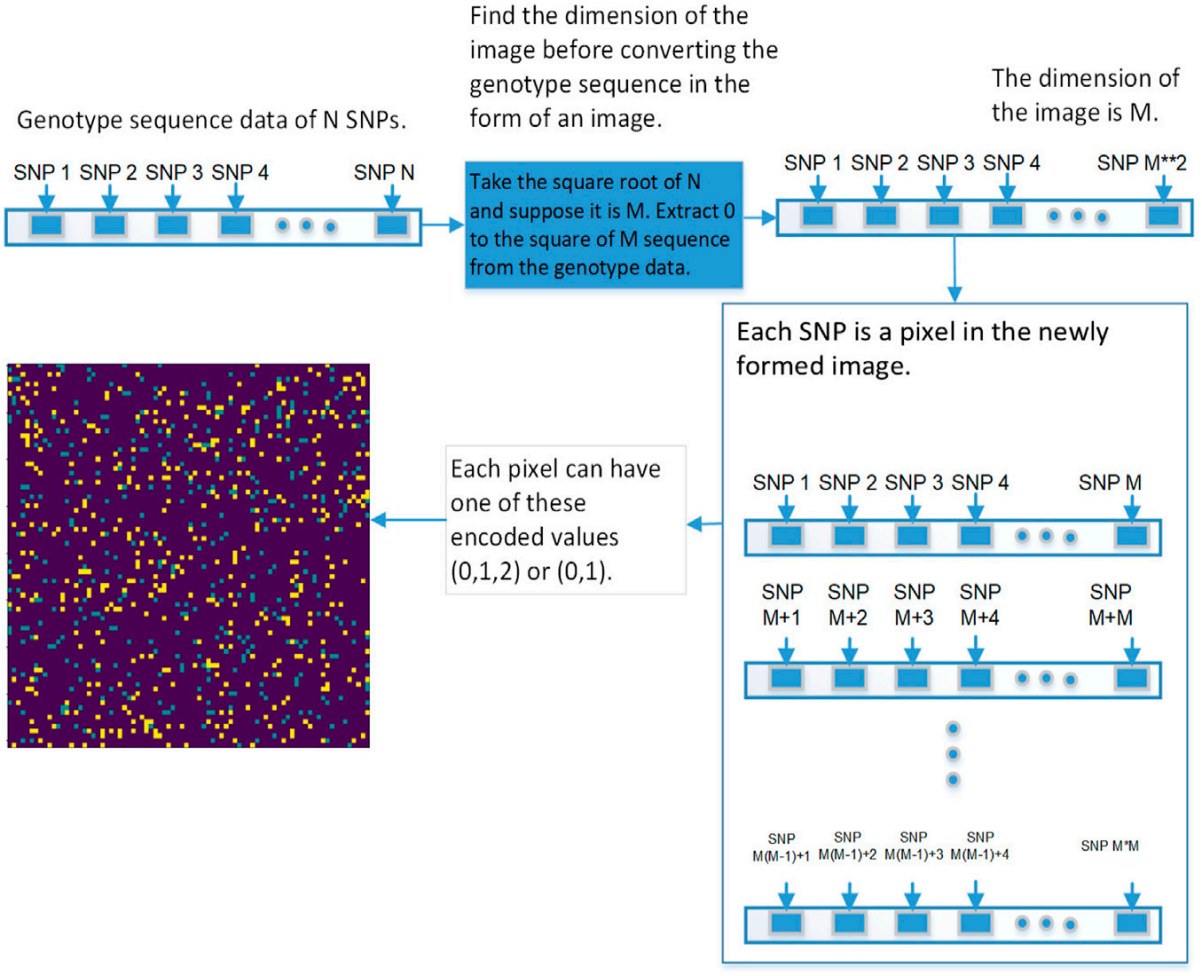
This diagram shows the first approach of converting the genetic sequence of SNPs into an image.

**FIGURE 6:**
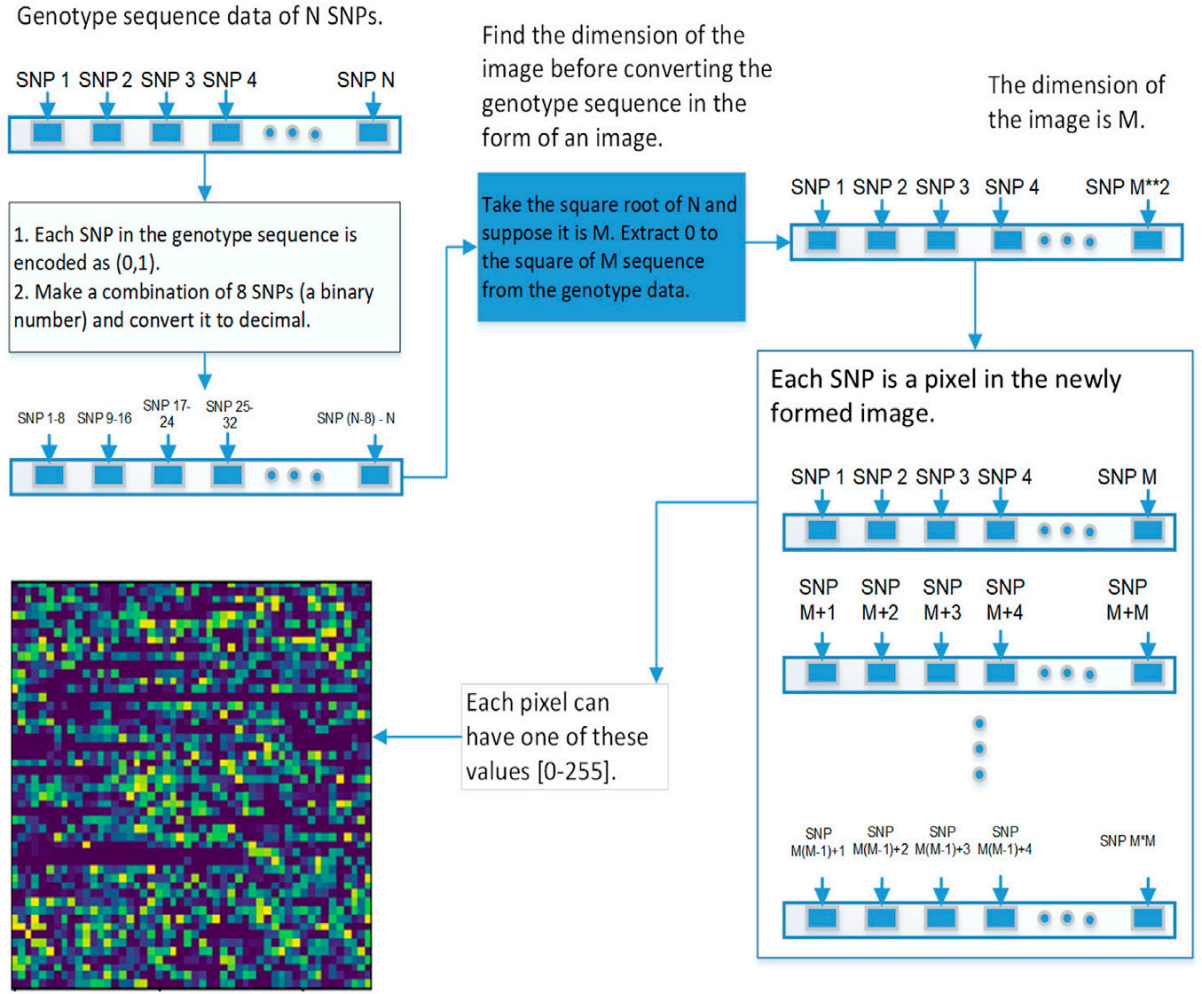
This diagram shows the second approach of converting the genetic sequence of SNPs into an image.

The first step is to convert genotype data into a square image. Genotype data is represented as [1⋯*N*], *N* is the size of genotype data, image dimension is represented as $\left\lfloor \sqrt[{2}]{N}\left\lfloor =M\right.\right.$ , extract [1⋯*M* ^2^] SNPs from genotype data, and present it in the form of the square 2D image. Each pixel represents data for a specific SNP, which can be represented in various ways. The first is the binary encoding scheme in which genotype is respensted in this way (aa = 0, aA//Aa = 1, AA = 0) (See Figure 5). In second encoding scheme genotype data is respensted in this way (aa = 0, aA//Aa = 1, AA = 2) (See Figure 6). In the third encoding scheme, genotype data is represented in the form of an image having a pixel range from 0 to 255. First the genotype data is converted to binary encoding (aa = 0, aA//Aa = 1, AA = 0), after that set of 8 SNPs are combined to form a pixel having range 0–255. This encoding scheme can incorporate a lot of genotype information, but it did not work, so we removed this one.

#### 3.5.3 Finding Image Similarity

Even if we see CNN, it tries to find similar images that fall in a particular category using a specific pattern found in an image. When it comes to discovering how similar two images are, then there are many things we have to consider. For instance, the window size of the sequence we want a comparison. The second is the evaluation metrics. In the case of genotype images, the similarity is measured pixel by pixel or SNP by SNP. This will shift the problem from image classification to image similarity calculation. Evaluation metrics like Mean Squared Error, Root Mean Squared Error, and Spatial Correlation Coefficient can be used to find the similarity between two images. We can also visualize the longest common sequence between two genotype files. In one dimension, it would not be easy to visualize, but when genotype data is in the form of images, we can easily visualize the longest common sequence between images, which is also a type of analysis in genomics.

#### 3.5.4 2DCNN Model Visualization

**FIGURE 7:**
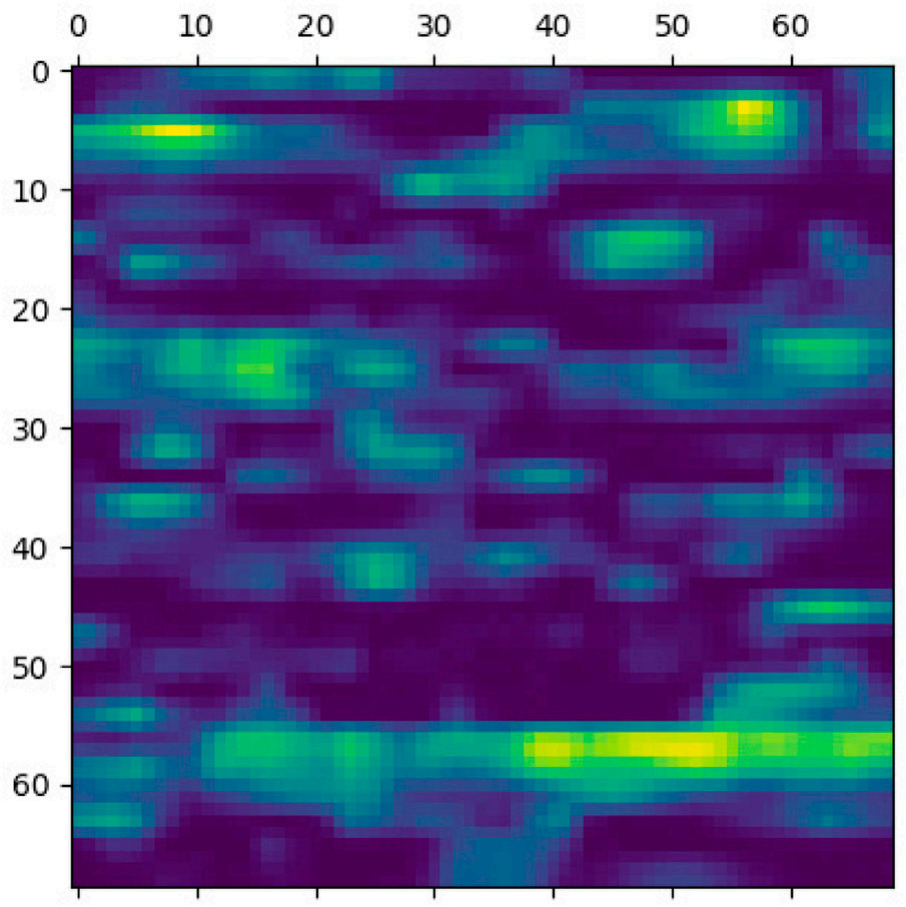
Classification region for a sample case.

**FIGURE 8:**
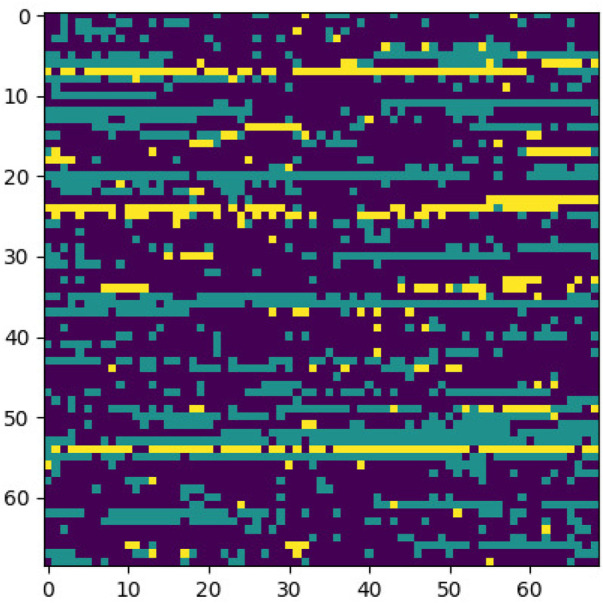
Sample case generated through encoding 2 and p-value = 5e-30.

**FIGURE 9:**
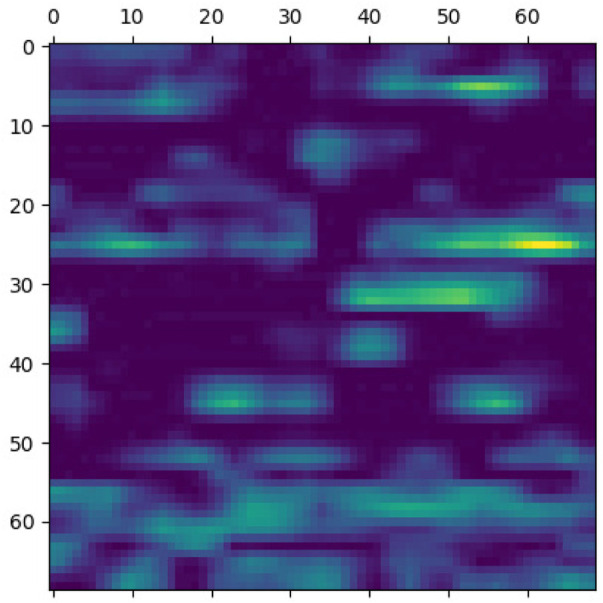
Classification region for a sample control.

**FIGURE 10:**
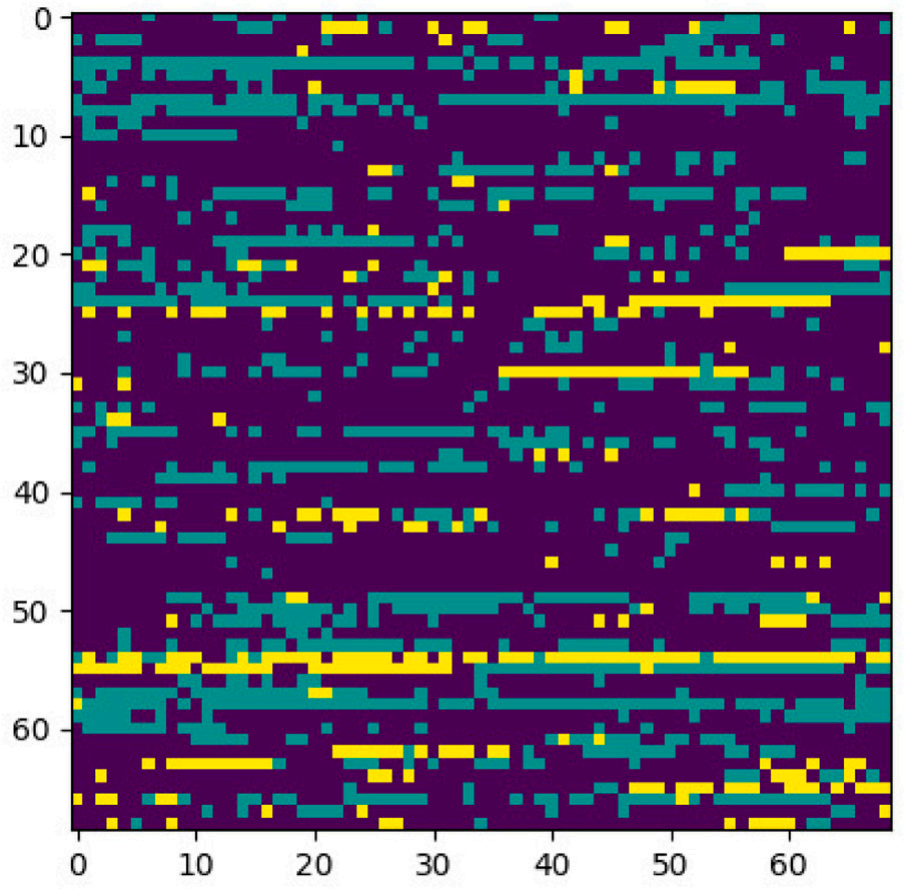
Sample control generated through encoding 2 and p-value = 5e-30.

This section elaborates on the reasons for using the 2DCNN algorithm. 2DCNN algorithm uses two-dimensional images, and it can help us to visualize what the deep learning model is learning, known as filter visualization. Secondly, it helps to identify the regions which cause a particular person to fall in the case or control category, known as classification region visualization (See Figures 7–10).

Identifying the classification region is one of the applications of the proposed method but is related to image segmentation rather than image classification. In an object classification problem (apples vs orange), image segmentation can find the area in an image where a particular object exists, but in the case of genetic images, that will not work. So, rather than image segmentation, filter visualization can help find the area that makes a particular person a case or control. The highlighted area contains SNPs (which can have a linear or non-linear relationship) whose values make a particular person a case or control in terms of biological interpretation. This is what the machine learning model tries to learn. This is also done in 1DCNN, but we cannot visualize the classification region in 1DCNN. Using 2DCNN gives us this advantage.

## 4 Results

This section elaborates on the result of using 2DCNN on genotype data. Tables 3, 4 show the average of all iterations of the 2DCNN and 1DCNN, respectively. The best test accuracy was 0.82 (p-value = 5.05e-30) for 2DCNN and 0.85 (p-value = 5.05e-50) for 1DCNN respectively. Even a two-dimensional convolution neural network worked fine on genotype sequence, yielding accuracy comparable to 1DCNN.

**TABLE 3:** 2DCNN: Average accuracies of all iteration.

*p*-values	E1–Training Accuracy	E1–Validation Accuracy	E1–Test Accuracy	E2–Training Accuracy	E2–Validation Accuracy	E2–Test Accuracy	Number of SNPs
pv_1.0	0.502	0.5	0.506	0.502	0.5	0.504	12,631
pv_5.05915e-10	0.57	0.566	0.564	0.504	0.502	0.504	3351.4
pv_5.05915e-30	**0.86**	**0.818**	**0.824**	0.654	0.63	0.64	603
pv_5.05915e-50	0.844	0.816	0.824	0.756	0.736	0.74	148.8

Bold text represents the best results for a particular *p*-value threshold.

**TABLE 4:** 1DCNN: Average accuracies of all iteration.

*p*-values	Training Accuracy	Validation Accuracy	Test Accuracy
pv_1.0	0.506	0.49	0.502
pv_5.05915e-10	0.67116	0.65144	0.648
pv_5.05915e-30	0.83164	0.798	0.79
pv_5.05915e-50	**0.89316**	**0.848**	**0.854**

Bold text represents the best results for a particular *p*-value threshold.

Results for 2DCNN are represented in this format: The first column shows the p-value threshold. The 2nd, 3rd, and 4th columns show training, validation, and test accuracies for encoding scheme 1. The 5th, 6th, and 7th columns show the training, validation, and test accuracy for encoding scheme 2. We restricted the results to only the first two encodings. The last column shows the number of SNPs included in each p-value threshold. E1 means encoding scheme 1 (aa = 0, aA//Aa = 1, AA = 2), and E2 (Figure 6) means encoding scheme 2 (aa = 0, aA//Aa = 1, AA = 0). Results for 1DCNN are represented in this format: The first column shows the p-value threshold. The 2nd, 3rd, and 4th columns show training, validation, and test accuracies.

**FIGURE 11:**
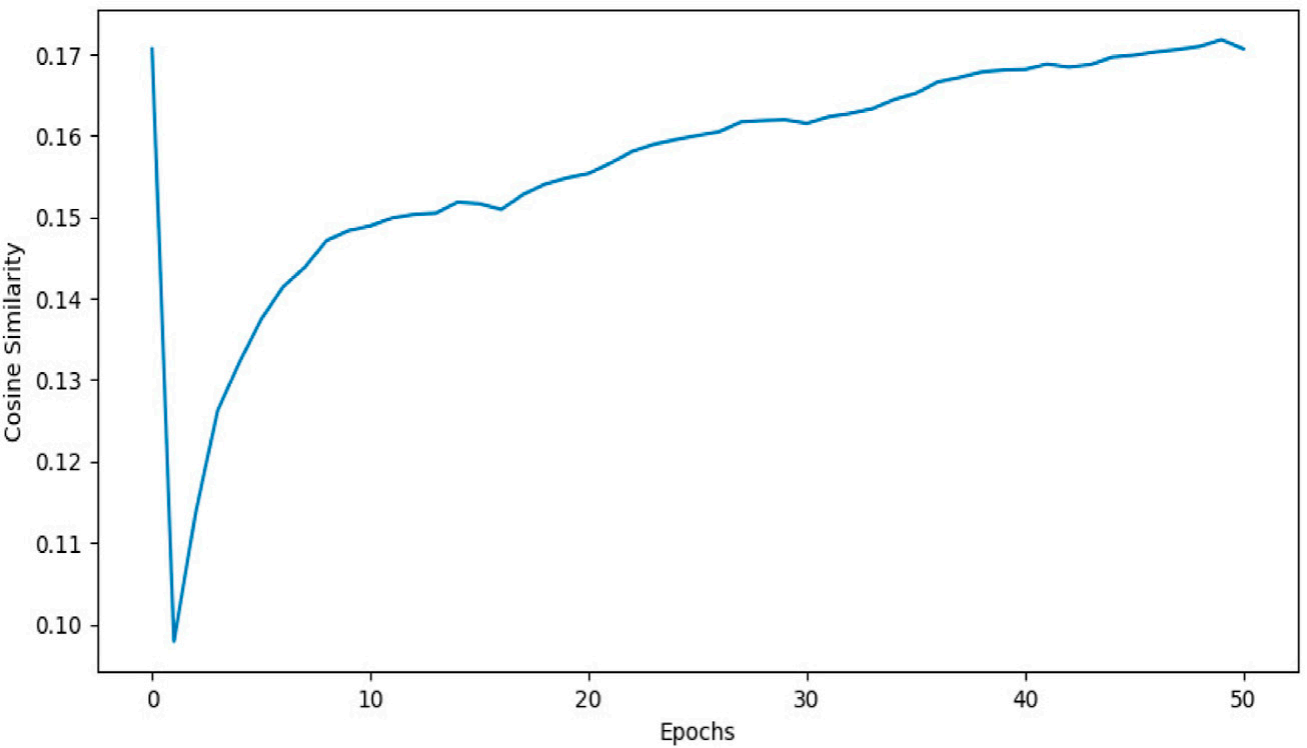
This figure shows the cosine similarity between the second last layer of the 1DCNN and 2DCNN model. 1DCNN model is trained and saved. The 2DCNN model is trained, and the similarity between the weights of the 2DCNN model and the 1DCNN model is calculated at each epoch. As we increase the number of epochs, the similarity between weights increases.

1DCNN (p-value = 5.0 e-50) yields an accuracy of 0.84 and 2DCNN (p-value = 5.0 e-30) yields an accuracy of 0.82. Interestingly, different algorithms give almost the same result, and there can be multiple explanations for that. Though both algorithms yield the same results, 2DCNN helps visualize patterns in the images. To investigate the similarity between the algorithms, we calculated the similarity between the second last layer of 1DCNN and 2DCNN models as shown in Figure 11. It is not possible to compare the convolutional layer because both models have a different number of parameters, but the second last, fully connected layer contains ten neurons in both models.

## 5 Conclusion

The manuscript illustrated the use of 2DCNN for case/control classification on genotype data by converting genotype data into images. We compared the results of 2DCNN with those of 1DCNN and noticed that there was not much difference between the final accuracies of the two algorithms. Second, we noticed that the training/test accuracies for the five iterations generated using 2DCNN had less variation than 1DCNN, suggesting that 2DCNN was more stable than 1DCNN.

Suppose 2DCNN algorithms, originally designed for image classification, can work for genotype data. In that case, algorithms from other research paradigms, such as transformers (for document classification), can also be used for genotype-phenotype classification. However, this may raise the question of biological interpretation of the results generated using such cross-field algorithms. This article aims to derive the research community’s attention to explore the encoding schemes for genotype data and machine learning algorithms that can be used on genotype data by changing the representation of the genotype data.

There are a few limitations associated with the proposed approach. First, we worked on the genotype data, and the inclusion of environment-related variables like covariates requires changes in the model’s architecture. So 2DCNN should be combined with the regular ANN model for the prediction. Second, the proposed approach may not work for images of any dimension, as shown in the results (See Supplementary Material).

## Data Availability

The original contributions presented in the study are included in the article/Supplementary Material, further inquiries can be directed to the corresponding author.
