# Supplementary material for: Can We Convert Genotype Sequences Into Images for Cases/Controls Classification?
Source: Front Bioinform. 2022 Jun 28;2:914435. doi: 10.3389/fbinf.2022.914435 (PMC9580854; doi:10.3389/fbinf.2022.914435)
Supplement: Supplementary file 1 [file DataSheet1.PDF]

Supplementary Material

1 SUPPLEMENTARY DATA

This section contains supplementary information for both datasets and the encoded images of the first sample of the HapMap3 data. Tables S1, S2 ,S3, S4, and S5 list the results of the 2DCNN for five iterations. Tables S6, S7, S8, S9, and S10 list the results of the 1DCNN for the five iterations. Figures S1, S2, and S3 show the representation of the three encodings for each chromosome of the first sample of HapMap3 data. Figures S4 and S5 show the filter visualization for the first and second layers of the 2DCNN model showing the regions (which make a particular person a case or control) in the images are identified as the input is passed from the top to bottom layer.

2 SUPPLEMENTARY TABLES AND FIGURES

P-values	E1 - Training Accuracy	E1 - Validation Accuracy	E1 - Test Accuracy	E2 - Training Accuracy	E2 - Validation Accuracy	E2 - Test Accuracy	Number of SNPs
**pv_1.0**	0.5	0.49	0.51	0.5	0.5	0.51	12631
**pv_5.05915e-10**	0.5	0.49	0.51	0.5	0.51	0.51	3343
**pv_5.05915e-30**	**0.87**	**0.8**	**0.82**	0.5	0.5	0.5	596
**pv_5.05915e-50**	0.83	0.8	0.81	0.84	0.81	0.81	145

Table S1. 2DCNN: Iteration 1.

P-values	E1 - Training Accuracy	E1 - Validation Accuracy	E1 - Test Accuracy	E2 - Training Accuracy	E2 - Validation Accuracy	E2 - Test Accuracy	Number of SNPs
**pv_1.0**	0.5	0.51	0.5	0.5	0.5	0.5	12631
**pv_5.05915e-10**	0.84	0.84	0.8	0.51	0.49	0.5	3347
**pv_5.05915e-30**	**0.89**	**0.86**	**0.85**	0.51	0.49	0.5	603
**pv_5.05915e-50**	0.85	0.82	0.82	0.78	0.75	0.76	152

Table S2. 2DCNN: Iteration 2.

P-values	E1 - Training Accuracy	E1 - Validation Accuracy	E1 - Test Accuracy	E2 - Training Accuracy	E2 - Validation Accuracy	E2 - Test Accuracy	Number of SNPs
**pv_1.0**	0.5	0.48	0.51	0.5	0.49	0.51	12631
**pv_5.05915e-10**	0.5	0.51	0.51	0.5	0.5	0.51	3323
**pv_5.05915e-30**	**0.87**	**0.83**	**0.85**	0.5	0.5	0.5	601
**pv_5.05915e-50**	0.85	0.83	0.85	0.8	0.78	0.79	141

Table S3. 2DCNN: Iteration 3.

.

2.1 Figures

P-values	E1 - Training Accuracy	E1 - Validation Accuracy	E1 - Test Accuracy	E2 - Training Accuracy	E2 - Validation Accuracy	E2 - Test Accuracy	Number of SNPs
**pv_1.0**	0.5	0.52	0.51	0.5	0.51	0.5	12631
**pv_5.05915e-10**	0.5	0.5	0.5	0.5	0.52	0.5	3361
**pv_5.05915e-30**	**0.83**	**0.8**	**0.79**	0.88	0.83	0.85	621
**pv_5.05915e-50**	0.84	0.81	0.82	0.86	0.83	0.84	156

Table S4. 2DCNN: Iteration 4.

P-values	E1 - Training Accuracy	E1 - Validation Accuracy	E1 - Test Accuracy	E2 - Training Accuracy	E2 - Validation Accuracy	E2 - Test Accuracy	Number of SNPs
**pv_1.0**	0.51	0.5	0.5	0.51	0.5	0.5	12631
**pv_5.05915e-10**	0.51	0.49	0.5	0.51	0.49	0.5	3383
**pv_5.05915e-30**	**0.84**	**0.8**	**0.81**	0.88	0.83	0.85	594
**pv_5.05915e-50**	0.85	0.82	0.82	0.5	0.51	0.5	150

Table S5. 2DCNN: Iteration 5.

P-values	Training Accuracy	Validation Accuracy	Test Accuracy
**pv_1.0**	0.5	0.5	0.5
**pv_5.05915e-10**	0.5	0.5	0.5
**pv_5.05915e-30**	0.92	0.88	0.87
**pv_5.05915e-50**	0.8823	0.82	0.82

Table S6. 1DCNN: Iteration 1.

P-values	Training Accuracy	Validation Accuracy	Test Accuracy
**pv_1.0**	0.51	0.49	0.5
**pv_5.05915e-10**	0.5	0.5	0.5
**pv_5.05915e-30**	0.5	0.5	0.5
**pv_5.05915e-50**	0.89	0.85	0.86

Table S7. 2DCNN: Iteration 2.

P-values	Training Accuracy	Validation Accuracy	Test Accuracy
**pv_1.0**	0.5	0.5	0.5
**pv_5.05915e-10**	0.9458	0.8972	0.9
**pv_5.05915e-30**	0.8982	0.85	0.86
**pv_5.05915e-50**	0.9035	0.88	0.89

Table S8. 2DCNN: Iteration 3.

P-values	Training Accuracy	Validation Accuracy	Test Accuracy
**pv_1.0**	0.51	0.5	0.5
**pv_5.05915e-10**	0.5	0.5	0.5
**pv_5.05915e-30**	0.91	0.87	0.86
**pv_5.05915e-50**	0.91	0.87	0.85

Table S9. 2DCNN: Iteration 4.

P-values	Training Accuracy	Validation Accuracy	Test Accuracy
**pv_1.0**	0.51	0.46	0.51
**pv_5.05915e-10**	0.91	0.86	0.84
**pv_5.05915e-30**	0.93	0.89	0.86
**pv_5.05915e-50**	0.88	0.82	0.85

Table S10. 2DCNN: Iteration 5.

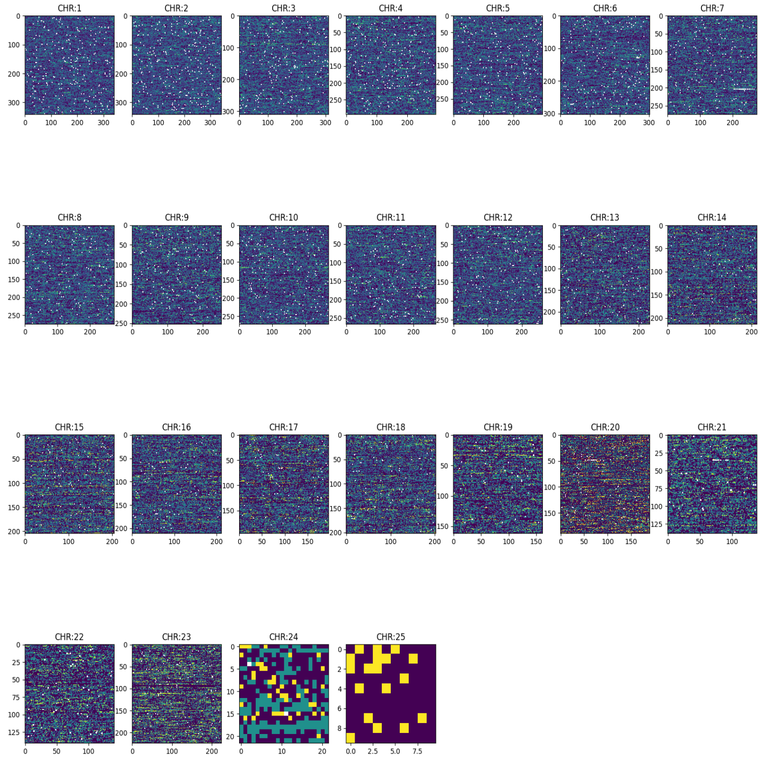

Figure S1. This diagram shows the first encoding scheme for all chromosomes.

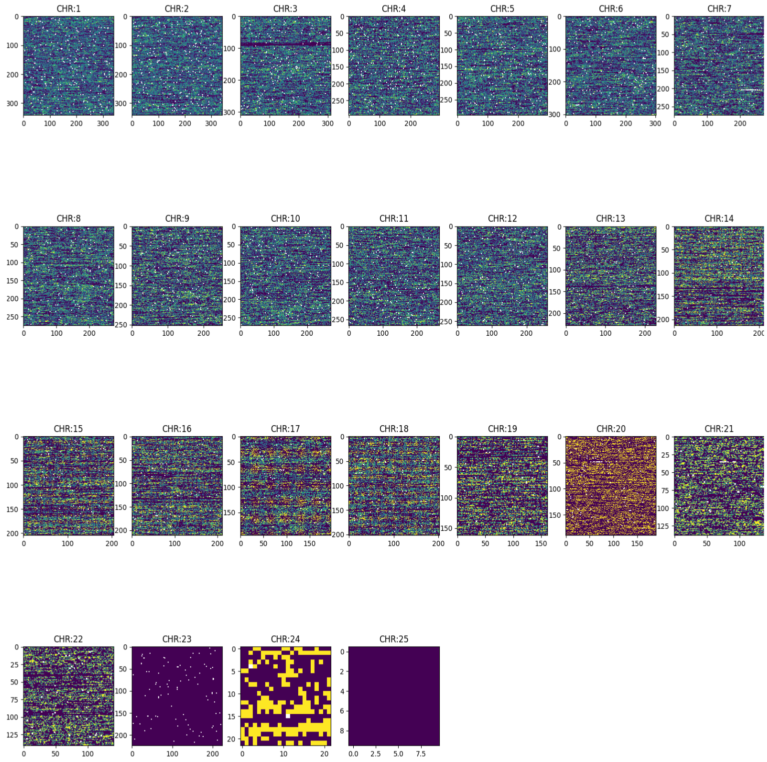

Figure S2. This diagram shows the second encoding scheme for all chromosomes.

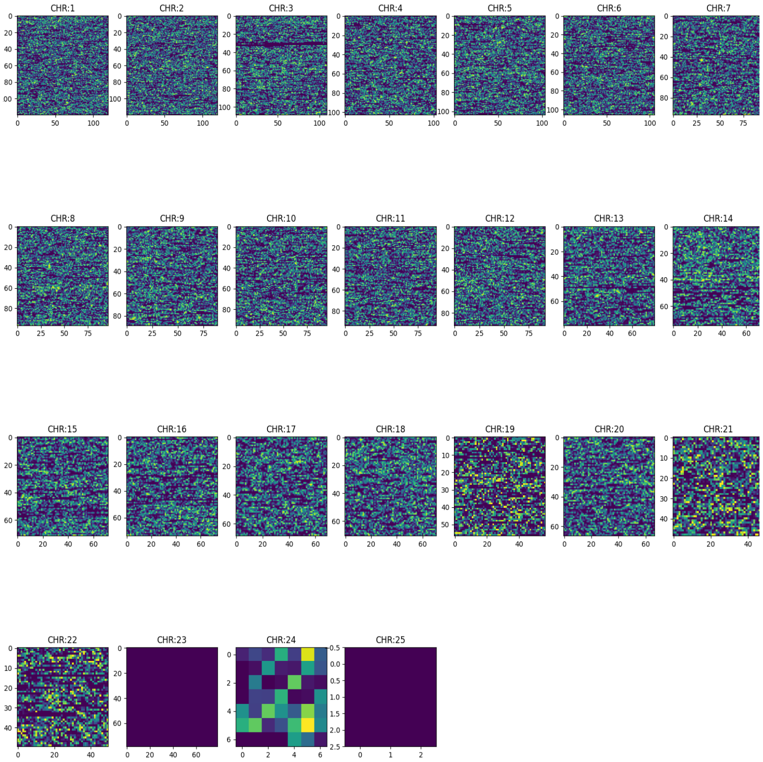

Figure S3. This diagram shows the third encoding scheme for all chromosomes.

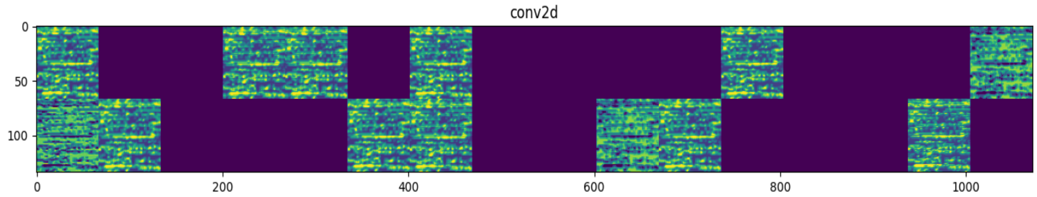

Figure S4. Filter visualization of first convolution layer. There are 32 filters, and each row contains 16 filters. Some filters found useful information from the genetic image, and the remaining are blank.

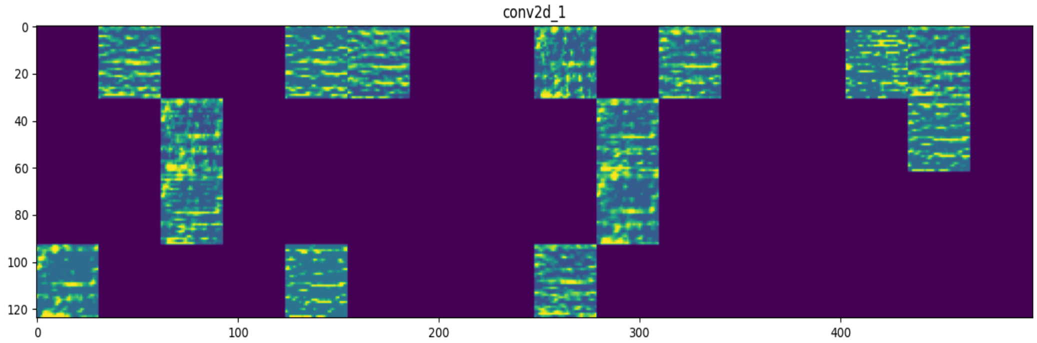

Figure S5. Filter visualization of second convolution layer. There are 64 filters, and each row contains 16 filters. Some filters found useful information from the genetic image, and the remaining are blank.
